# Toward noninvasive monitoring of ongoing electrical activity of human uterus and fetal heart and brain

**DOI:** 10.1016/j.clinph.2017.08.026

**Published:** 2017-12

**Authors:** S. Lew, M.S. Hämäläinen, Y. Okada

**Affiliations:** a Division of Newborn Medicine, Department of Medicine, Boston Children’s Hospital and Harvard Medical School, Boston, MA 02115, USA; b Athinoula A. Martinos Center for Biomedical Imaging, Department of Radiology, Massachusetts General Hospital and Harvard Medical School, Boston, MA 02119, USA

**Keywords:** Prenatal medicine, Magnetocardiography (MCG), Electrocardiography (ECG), Electrohysterography (EHG), Magnetoencephalography (MEG), Electroencephalography (EEG)

## Abstract

**Objective:**

To evaluate whether a full-coverage fetal-maternal scanner can noninvasively monitor ongoing electrophysiological activity of maternal and fetal organs.

**Methods:**

A simulation study was carried out for a scanner with an array of magnetic field sensors placed all around the torso from the chest to the hip within a horizontal magnetic shielding enclosure. The magnetic fields from internal organs and an external noise source were computed for a pregnant woman with a 35-week old fetus. Signal processing methods were used to reject the external and internal interferences, to visualize uterine activity, and to detect activity of fetal heart and brain.

**Results:**

External interference was reduced by a factor of 1000, sufficient for detecting signals from internal organs when combined with passive and active shielding. The scanner rejects internal interferences better than partial-coverage arrays. It can be used to estimate currents around the uterus. It clearly detects spontaneous activity from the fetal heart and brain without averaging and weaker evoked brain activity at all fetal head positions after averaging.

**Conclusion:**

The simulated device will be able to monitor the ongoing activity of the fetal and maternal organs.

**Significance:**

This type of scanner may become a novel tool in fetal medicine.

## Highlights

• Evaluated a fetal-maternal scanner for monitoring electrical maternal and fetal organ activity.
• The simulated scanner can monitor the uterine and fetal heart and brain activity online.
• Biomagnetic monitors similar to this instrument should be useful in clinical neurophysiology.

## 1 Introduction

We still know relatively little about the electrophysiology of the human uterus and the fetal heart and brain during pregnancy. Magnetic Resonance Imaging (MRI) and ultrasound are useful for anatomical imaging of the maternal and fetal organs (Reddy et al., 2008, Studholme, 2011). Ultrasound can also be used to monitor uterine and fetal heart contractions and movement of the valves as well as blood flow in the heart (Tonni et al., 2015). However, sensitive techniques to measure the ongoing electrophysiological activity of the uterus and fetal heart and brain need further development.

Electrical potential measurement techniques can be used to monitor the activity of maternal heart and other organs. The contractions of the uterus can be measured mechanically (tocodynamometry - Bakker et al., 2010) and its electrical activity can be measured with electrohysterogram (EHG) (Alamedine et al., 2013, Alexandersson et al., 2015). However, this field is still in its infancy and we know relatively little of where the contraction may be initiated and how it propagates during preterm and normal labor. The physiology during the quiescent period before the onset of labor is even less understood.

Measurement of the electrical activity of the human fetal organs is more difficult. Fetal electrocardiogram (fECG) can be monitored noninvasively on the maternal abdominal surface (Taylor et al., 2003, Nii et al., 2006, Gardiner et al., 2007). The QRS components can be measured clearly in real time, but the P and T waves are often difficult to detect without averaging across 10–100 beats. Due to the relatively poor signal quality and difficulty of obtaining reliable measurements, this method is not routinely used to monitor fetal heart activity. Fetal electroencephalographic (fEEG) signals measured on the abdominal wall are even weaker than fECG and thus too difficult to detect reliably and not useful clinically.

Electrical currents in the fetal organs also produce magnetic fields detectable outside the maternal torso. It has been recognized that the biomagnetic techniques are better suited for noninvasive measurements of physiological conditions of the fetus and mother (Wakai et al., 1994, Eswaran et al., 2007, Sheridan et al., 2010) because the spatial spread and smearing of surface potentials are in large part avoided by measuring the activity magnetically. The magnetic field is not significantly attenuated by the *vernix caseosa* (Wakai et al., 2000), which is known to significantly reduce the fECG during 20–36 weeks of GA (Taylor et al., 2003). The amniotic fluid and the adipose tissues do not affect the component of the magnetic field normal to the abdominal surface as much as the surface potential (Peters et al., 2005). Advanced instruments are available today (e.g. Vrba et al., 2004) to detect the signals from the uterus (Eswaran et al., 2002) and from the fetal heart (fetal magnetocardiography - fetal MCG: Wakai et al., 1994) and brain (fetal magnetoencephalography - fetal MEG: Eswaran et al., 2007). Fetal MCG, in particular, is becoming recognized as a useful clinical tool for detecting fatal cardiac activity during the mechanically silent period that cannot be detected with echocardiography (Wiggins et al., 2013, Donofrio et al., 2014).

Nevertheless, the magnetic signals of ongoing spontaneous activity from these organs, especially the fetal brain, are not routinely measured even with the advanced magnetic field sensor arrays. The analysis requires a considerable amount of effort in signal preprocessing, due to heavy contamination from the spatial mixing of the signals from different internal organs and external noise sources. The relatively poor SNR, especially for fMCG and fMEG, has prevented the routine monitoring of their ongoing activity in real time. These problems, in addition to the cost of constructing such a facility, have considerably limited widespread acceptance of biomagnetic instruments in obstetrics and fetal medicine.

We tested an idea that the quality of biomagnetic signals can be improved significantly by using a full-coverage, whole-body sensor array instead of a partial-coverage sensor array as in existing biomagnetic instruments. This idea is based on our experience in rejecting external magnetic field noise sources for the whole-head 375-channel pediatric MEG system (“babyMEG”) we have developed recently (Okada et al., 2016). For the babyMEG, it is possible to reject the external noise detected by the sensors located inside a magnetic shielding enclosure, even though the sensors are all magnetometers, and to clearly detect spontaneous brain signals on a monitor online. We hypothesized that this full-coverage provides a significant improvement in rejecting both the external and internal noise sources, thereby making it possible to routinely monitor the ongoing activity of the maternal and fetal organs using a fetal-maternal (FM) scanner similar in design to the one used in this simulation study. Waldert et al. (2007) have already shown that it is possible to monitor the fMCG in real time using a partial-coverage biomagnetometer. We believe that it will become possible to simultaneously measure the spontaneous activity of not only the fetal heart, but also the other organs including the fetal brain, and display their signals on a monitor online. Such a capability could significantly advance the field of obstetrics and fetal medicine since the real-time information will provide immediate feedback in the condition of the mother and fetus that could be useful in improving the monitoring procedure or intervention. The results of our simulation analysis support this hypothesis.

## 2 Methods

This section describes our method for constructing the realistic torso model and several types of sensor array used in our simulation study. We present evaluation of an eventual FM scanner with a full-coverage sensor array wrapped around the torso of a pregnant woman to help readers understand the methods we evaluated for rejecting the interference from external noise sources and internal organs and how we determined the anticipated performance level of a full-coverage sensor array.

### 2.1 Realistic model of the torso and computation of biomagnetic fields

**Fig. 1:**
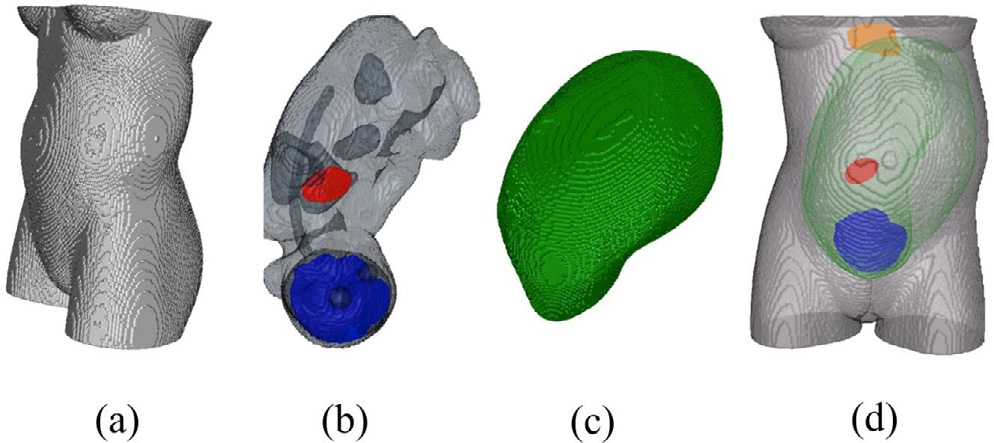
The compartments of the FEM model: (a) torso (b) fetus brain (blue), eyes, heart (red), lungs, stomach, bladder, (c) uterus, (d) torso with fetus brain, fetus heart, uterus, and mother heart (orange). (For interpretation of the references to colour in this figure legend, the reader is referred to the web version of this article.)

We constructed a realistic model of the torso of a pregnant woman carrying a fetus of 35 week GA based on the segmented anatomical data provided by FEMONUM repository (Bibin et al., 2010) (Fig. 1). These data consist of fetal heart, lungs, eyes, bladder, stomach, brain, uterus, umbilical cord, amniotic fluid, and mother torso. We used a Finite Element Method (FEM) to represent the torso with its internal organs (Lew et al., 2013). The FEM model represents the torso by 2,462,338 hexahedral elements (each 2 × 2 × 2 mm in size) and 2,540,065 nodes, generated by a geometry-adapted meshing algorithm. The FEM model allows accurate calculation of the magnetic field generated by electric currents produced in each organ of interest, by taking into account the realistic geometry and boundary surfaces separating regions of differing electrical conductivity. To compute the magnetic field, we used the open-source SimBio-NeuroFEM software package (SimBio, 2012).

### 2.2 Partial- and full-coverage magnetic field sensor arrays

**Fig. 2:**
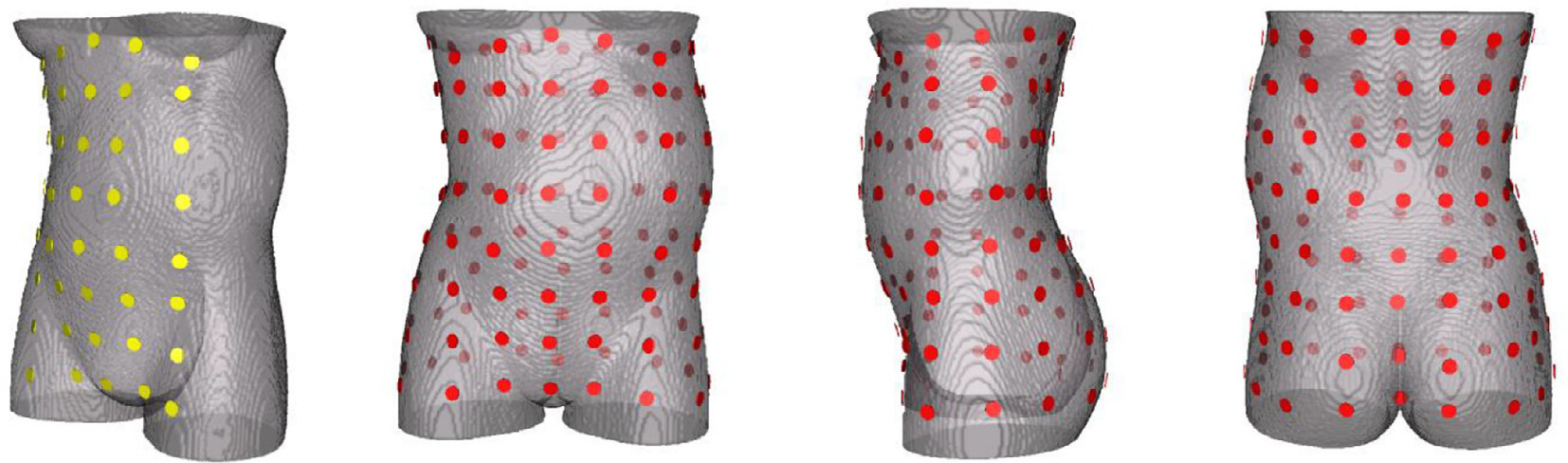
(Left) Partial-coverage sensor array (8 × 5 array, 40 channels). (Right) Full-coverage sensor array (8 × 16 array, 128 channels).

We computed the magnetic fields produced by external noise sources and different internal organs for two different types of sensor array. Fig. 2, left, shows an example of the partial-coverage array with a 120° coverage consisting of 5 × 8 sensors, 40 sensors total, on the anterior side of the torso of the pregnant woman. This array covers the abdominal surface similar to the 151-channel biomagnetometer called SARA (SQUID array for reproductive assessment) (Vrba et al., 2004), which is the most advanced FM scanner in use today. Although elegantly designed SARA provides a limited coverage over the abdominal surface. The partial coverage array in Fig. 2 extends over a longer distance up to the chest. In addition we designed partial-coverage sensor arrays with different degrees of angular coverage for studying the sensitivity of signal reconstruction to the area covered. The full-coverage array (Fig. 2, right) consists of 8 rings of 16 sensors each for a total of 128 sensors. Each sensor was assumed to be 5 mm in diameter, located ∼5 mm from the torso.

### 2.3 FM scanner

**Fig. 3:**
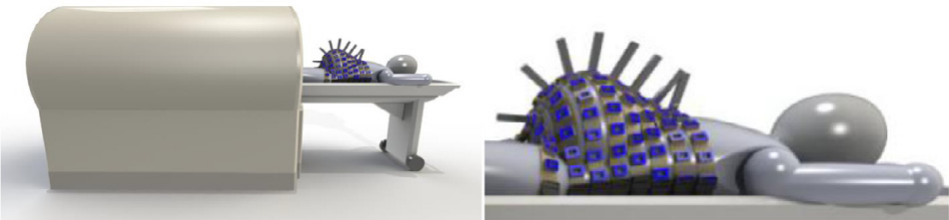
Initial design of the FM scanner with a full-coverage sensor array consisting of OPM sensors inserted into the light-weight, flexible belts placed around the body of the mother. Courtesy of Anthony Mascarenas, Tristan Technologies, Inc., San Diego, CA.

The initial design of our FM scanner is shown in Fig. 3. Although this scanner is still at the conceptual stage, the figure helps to visualize how the sensor array configurations of Fig. 2 may be used in practice. The sensors were modeled to be similar to the optically pumped miniature atomic magnetometers (OPMs) developed by Knappe and her colleagues (Griffith et al., 2010, Sander et al., 2012, Ledbetter et al., 2008), so that the results could apply to actual sensor arrays when such a scanner is constructed. Several groups have begun to use OPMs for biomagnetic measurements of the uterus and fetal heart (Wyllie et al., 2012, Shah and Wakai, 2013, Alem et al., 2015, Eswaran et al., 2017).

Unlike the SQUID sensors based on superconductivity, the OPMs operate at room temperature, eliminating the need for handling of a cryogen, thus making the instrument potentially more widely useful. In the actual array, the sensors will be placed in light-weight, flexible belts, separated by about 5 cm from each other. Each belt conforms to the shape and size of the torso of any pregnant woman, unlike the rigid, fixed-shape arrays in the existing biomagnetometers. This flexible array guarantees that the sensors are all placed at the closest possible distance of 5 mm above the skin, thereby providing the maximum possible SNR for these sensors. In actual testing, the mother would lie on a set of 8 belts on the bed. The belts are flexible and easily conform to the shape of the torso. Once the mother is on the bed, the rest of each belt is placed over the torso. Each OPM probe is very light and does not cause discomfort to the mother. Once the sensors are in place, the bed slides into the bore of the magnetic shield.

The shield will be made of two or three layers of hypermalloy metallic cylinders, combined with an external degaussing coil array and an internal active shielding coil array. The passive shielding creates an effectively “zero-field environment (<10 nT at DC and above up to about 100 Hz) for the zero-field OPMs to operate fully. The internal active shielding can be carried out by the low-sensitivity earth field OPMs (QTFA-00U, QuSpin) that can reduce the field further by a factor of about 1000 or to about 10 pT or less. This then brings the zero-field, high-sensitivity OPMs to operate within its dynamic range down to about 10 fT/√Hz.

As mentioned below, an additional noise cancellation software technique, based on SSP, can be used to reduce this magnetic field to levels sufficient for measuring the biomagnetic signals from the maternal and fetal organs. The detection cell at the bottom of each OPM probe senses the magnetic field over the abdomen with a sensitivity of 5–15 fT/√Hz comparable to that of SQUIDs (Griffith et al., 2010). The sensitivity assumed in our simulation is 10 fT/√Hz. This sensitivity is sufficient to measure the signals from the fetal brain as described in Results below.

Cross-talk is a phenomenon in which the magnetic field applied to each OPM probe for reduction of the static field and modulation of the OPM cell influences the signal sensed by the adjacent probes. In the present study we have assumed that there is no cross talk. We expect this assumption to be fairly accurate since the separation between the probes is approximately 5 cm. The cross-talk for actual OPMs probes fabricated by QuSpin is <1.6% for this geometry and their sensors (Vishal Shah, personal communication).

### 2.4 Rejection of external magnetic interference

The magnetic noise from external sources is a major factor deteriorating the SNR. The external magnetic field can be very strong compared to magnetic fields from internal organs. The DC field of the Earth is about 0.5 × 10^−4^ Tesla. The fluctuation of the ambient field is normally about ±1 μT, for example in a room in the main building of our Boston Children’s Hospital. This ambient magnetic field can be reduced by a magnetic shielding enclosure such as the one shown in Fig. 3. We expect the shielding enclosure can reduce the DC field to <10 nT, which is sufficiently low for the zero-field OPMs to operate. The internal active shielding will further reduce this noise to <10 pT (V Shah, QuSpin, Inc. personal communication). However, this level is still 1000 times greater than the noise of the OPMs.

**Fig. 4:**
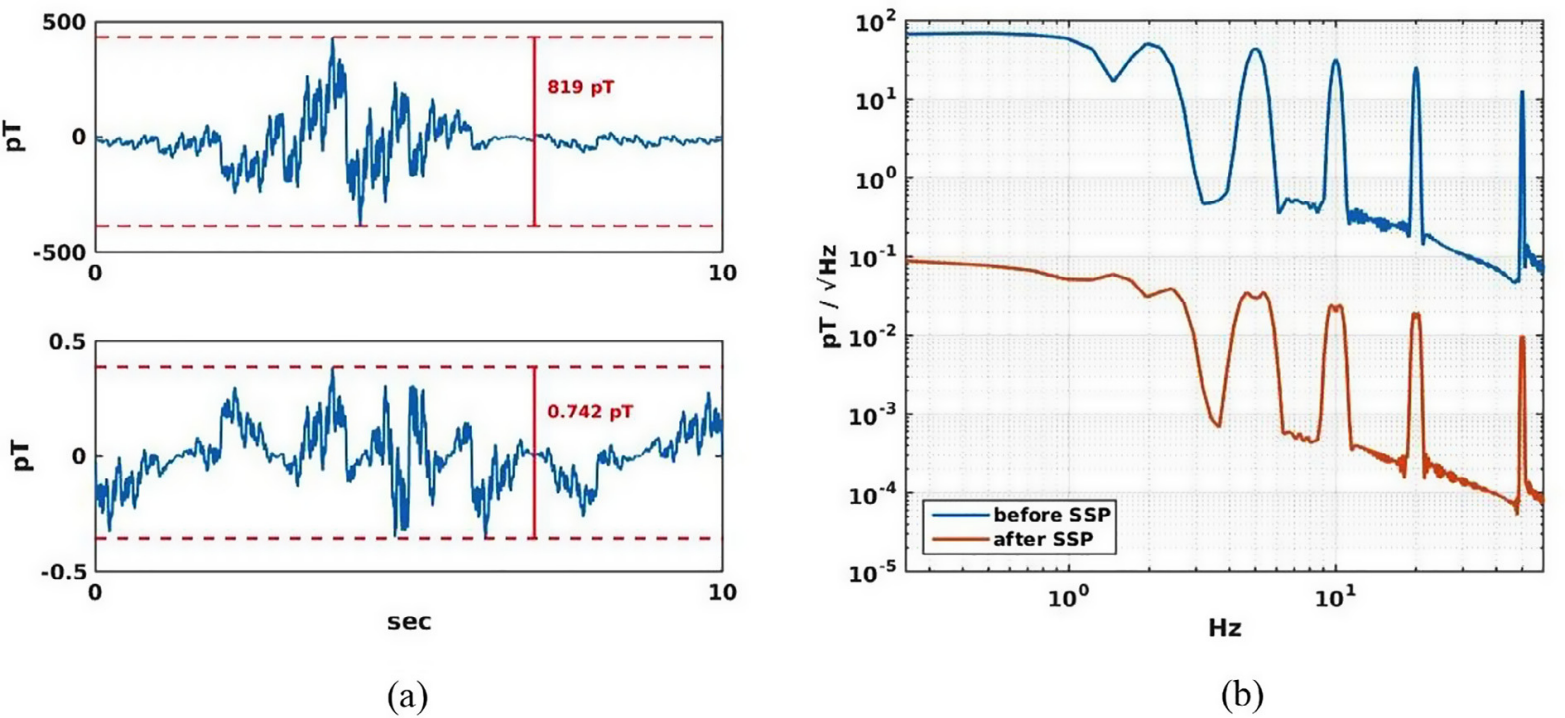
Rejection of external magnetic disturbance. (a) External noise field before (top) and after (bottom) SSP. (b) The same noise field presented in frequency domain.

Rejection of external interference is thus a major problem for operating an FM scanner. We evaluated how well this type of interference can be eliminated by a combination of a whole-body coverage and a noise cancellation software technique. An external magnetic noise source was simulated by a cart moving longitudinally on one side of the mother at a velocity of 1 m/s, 3 m away from the central longitudinal axis of the sensor array. A magnetic noise source placed on the cart produced a temporal waveform of 10 s in duration (see Fig. 4a, top right, for the waveform used), which consisted of sine waves of 0.1, 0.2, 0.5 1, 2, 5, 10, 20, and 50 Hz with varying amplitudes. The magnetic field due to the noise source was calculated for the full-coverage sensor array.

To reject the external magnetic noise, we used the signal space projection (SSP) method (Uusitalo and Ilmoniemi, 1997). SSP has been implemented, e.g., in a software (www.martinos.org/mne) developed by one of us (Hämäläinen) for MEG/EEG research. We hypothesized that the SSP can provide a significant noise rejection that is sufficient for online monitoring of the signals from the fetal organs when the field is measured with a full-coverage sensor array. The results shown below fully confirm this hypothesis.

### 2.5 Rejection of internal biological magnetic interference

**Fig. 5:**
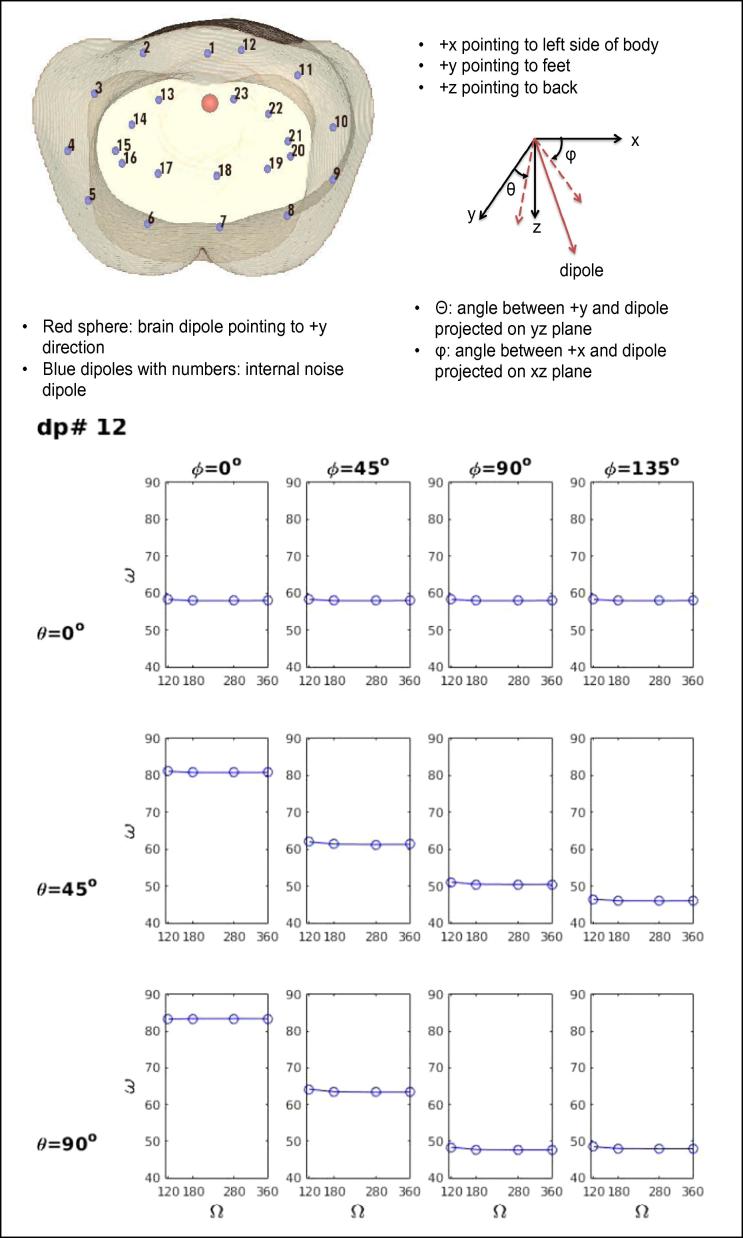
Subspace angle (ϖ) between the signal dipole (red sphere) and noise dipole 12 as a function of coverage angle of the sensor array (Ω) for different angular separations θ (angle between + y axis and the dipole projected on the yz plane) and ϕ (angle between + x and the dipole projected on xz plane). For this shallow noise dipole below the sensor array with the smallest coverage angle, ϖ is independent of Ω, but depends on θ and ϕ. Top inset shows the location of the signal dipole in the fetal brain (red sphere), pointing to feet, and 23 noise dipoles inside the torso.

**Fig. 6:**
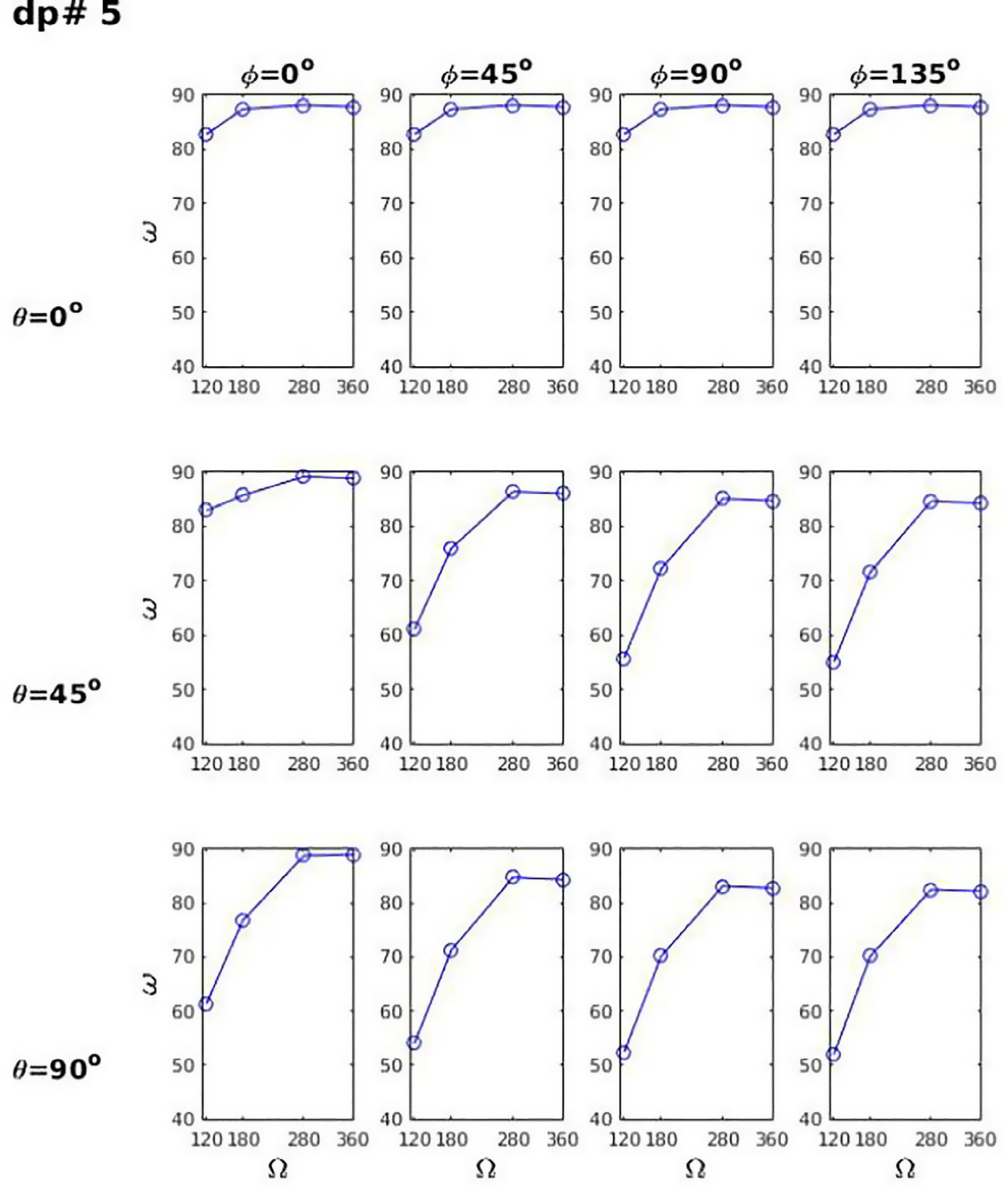
Subspace angle (ϖ) between the signal dipole (red sphere (see Fig. 5 inset)) and noise dipole 5 as a function of coverage angle of the sensor array (Ω) for different angular separations θ and ϕ. For this deep and lateral noise dipole, ϖ depends on Ω, θ and ϕ.

We also studied how our sensor array can remove contaminations from various internal noise sources. When one is interested in measuring the activity of the fetal heart or brain, the signal from this target organ is contaminated by the magnetic field from the internal organs of the mother that are nearby (stomach and intestines) or that surround these fetal organs (uterus and maternal abdominal muscles). We evaluated how well the full-coverage array can reject the noise from internal organs in comparison to various partial-coverage arrays. We placed 24 current dipoles inside the pregnant torso model by arranging two layers of 12 dipoles inside the body (see the inset in Fig. 5, Fig. 6). One dipole (shown by a red circle), 6 cm below the middle of the sensor array, was selected as a fetal brain source with the dipole directed toward the feet (+y axis). All other 23 dipoles were considered as internal noise sources. The orientation of each noise source was systematically varied away from the +y axis (θ = 0°, 45° and 90°) and from the +x axis (ϕ = 0–315° in steps of 45°). The magnetic field at the sensor array can be described by a signal-space vector for any dipole. The subspace angle (ϖ) is defined as the angle between the signal-space vector of interest and that of the noise. The magnetic field from a noise source can be attenuated more effectively without sacrificing the signal of interest as the subspace angle approaches 90° since the vectors then become more orthogonal and independent of each other. We studied the rejection of internal magnetic interference as a function of coverage angle (Ω) of the sensor array (120°, 180°, 280°, 360°).

### 2.6 Imaging active regions in the uterus

**Fig. 7:**
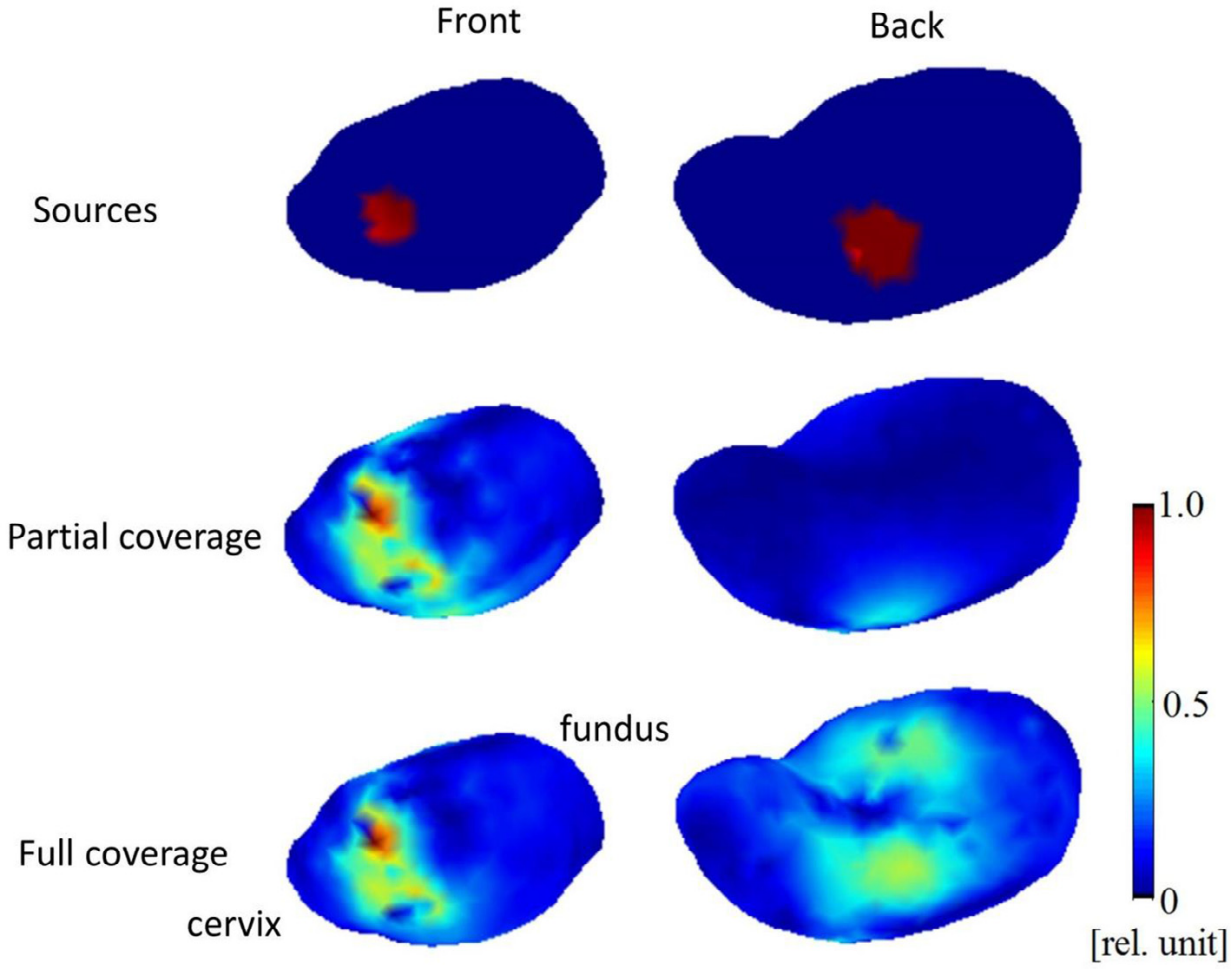
Imaging of the myogenic current in the entire uterus with the partial and full coverage sensor arrays.

Visualizing the electric activity over the entire uterine myometrial wall is valuable for understanding the electrophysiology during the quiescent period and the initiation and propagation of uterine contraction during preterm and term labor. Synchronization of activity in the myometrium could provide new insights into the nature of these processes. Thus, we tested whether the electrical activity in different regions of the uterus can be visualized based on magnetic field recordings using a partial-coverage array (5 × 8 sensors) and the full-coverage array (16 × 8 sensors). The outer surface of the myometrium in the FEM model with 873 nodes was defined as the source space. The activity was set up in a patch of the source space on the anterior side and in another patch on the posterior side of the uterus (Fig. 7, top row). The magnetic fields at the sensors of the two arrays were computed for these sources using the FEM model of the torso. To image the activity in the uterine wall, we used the minimum-norm estimate (MNE) (Hämäläinen and Ilmoniemi, 1994). The MNE has the minimum L2-norm among all current distributions that can explain the measured data. This method has been extensively used in EEG and MEG source localization and is available in several open-source software packages (Tadel et al., 2011, Gramfort et al., 2014) as well as in commercial software.

### 2.7 Calculation and detection of electrical activity of the fetal heart and brain

To evaluate the capabilities of the FM scanner for detecting the signals from internal organs, we modeled the activity in each target organ as an equivalent current dipole (ECD) source with a physiologically realistic amplitude and waveform. The source waveforms are based on the work of others. For the uterus, we used the data from the EHG database (Alexandersson et al., 2015) with a current dipole moment Q = 300 nAm which gives a field strength of 5 pT consistent with the range of magnitudes reported by (Tsukada et al., 1999). For the fetal heart, the waveform was adapted from fetal MCG with a Q = 650 nAm (Kandori et al., 1999). For the fetal brain, the waveform of spontaneous activity (trace discontinue) was adapted from fetal MEG (Eswaran et al., 2007) with a Q = 50 nAm.

**Fetal heart** - The detectability of electrical activity of the fetal heart was evaluated by first computing the magnetic fields produced by an external noise source, the uterus, the fetal heart and the fetal brain at the sensor locations of the full-coverage FM scanner and then by extracting the signal from the fetal heart in the presence of these “noise” sources. The magnetic field from the external noise source was calculated under the same condition as mentioned in Method section 4, except for the time course of the noise, which was a tapered 15 Hz sinusoid. The uterine dipoles were placed at five locations around the cervix of uterus. A single ECD was placed in the middle of the fetal heart and another dipole in the fetal brain. The magnetic field from the fetal heart was extracted from the mixture of the magnetic fields using a beamformer. In this method, data from all magnetic field sensors are combined to focus on the site of interest while suppressing interference from other potentially active sites. We used the linearly constrained minimum variance spatial filtering (LCMV) beamformer (Van Veen et al., 1997).

**Fetal brain (spontaneous activity)** - The detectability of electrical activity of the fetal brain was evaluated by following the same procedure used for extracting the magnetic field from the fetal heart. The same mixture of the magnetic fields from the different external and internal sources for the fetal heart was used for this analysis. The magnetic field from the fetal brain was extracted using the same beamformer as for the fetal heart. This procedure evaluated the detectability of spontaneous signals of the fetal brain.

**Fetal brain (evoked cortical activity)** - Cortical activity that can be evoked by external stimulations such as sound, light, or even linguistic stimuli is much weaker than spontaneous brain signals. The amplitudes are 10–100 fT_rms_ as compared to 100–500 fT_rms_ for spontaneous brain activity at the abdomen (spontaneous activity - Eswaran et al., 2007; evoked responses – Eswaran et al., 2005). To evaluate the detectability of evoked cortical signals we placed an ECD of 50 nAm in the fetal brain comparable to the dipole moments recorded by others (5–40 nAm: Nevalainen et al., 2015, Pihko et al., 2009). The dipole was placed at four different locations, 22, 62, 102, and 142 mm deep from the anterior torso surface, to cover the range of sensor-to-source distances during the pregnancy. The cosine-square shaped pulse was generated as the dipole waveform with 1 Hz frequency for 2 min. We calculated the magnetic signal that would be sensed by four types of sensor array (120°, 180°, 280° and 360°) with and without internal noise sources. The internal noise sources included the magnetic field from the maternal heart, stomach, uterus, and small and large intestines and fetal heart with characteristic temporal waveforms. Their dipole moments were about 100 times larger than that of the fetal brain in order to evaluate how the internal noise can be reduced with the SSP.

### 2.8 Coregistration of maternal and fetal body and organs with the sensor array

The coregistration of the maternal and fetal body and organs with the sensor array is an important aspect in the implementation of any FM scanner as it is the case for any MEG or MCG measurements. In this paper, we will not devote much space on this issue since it will be addressed during the implementation of this type of instrument. Nevertheless, we wish to point out that the design of the sensor array shown in Fig. 3 does allow for simultaneous measurements of the fetal position and biomagnetic signals. One or more 2D ultrasound imagers can be placed between the belts for 3D imaging of the fetal position. Their outputs can be used to construct a 3D image of the fetus for the purpose of updating the coregistration every time the fetus moves in the womb.

## 3 Results

### 3.1 Rejection of external magnetic interference

Fig. 4a shows the waveforms and amplitudes of the external magnetic field at the sensor detecting maximum strength before (top) and after (bottom) applying the SSP. As described in Method, the waveform was a mixture of sinusoidal signals at 0.1, 0.2, 0.5 1, 2, 5, 10, 20, and 50 Hz with varying amplitudes. Its amplitude spectrum is shown in blue in Fig. 4b. The peak-to-peak noise is ∼800 pT in the time domain. The spectrum energy density is ∼100 pT/√ Hz up to about 1 Hz and the density decrease to 10 pT/√Hz at the 50 Hz peak. This is the noise level expected in the sensor array region after applying the passive and active shielding methods (V. Shah, QuSpin, Inc., personal communication). The trace in red in Fig. 4b is the spectrum after applying the SSP. The SSP removes low-dimensional noise subspace from the data before subsequent processing. We determined the noise subspace with Principal Component Analysis (PCA) of the noise covariance matrix. We included the first eight principal vectors to the noise subspace, approximately corresponding to the three orthogonal spatially uniform field components and the five independent field gradients. The maximum peak-to-peak amplitude of the noise was reduced from 819 pT to 0.74 pT. The SSP reduced the noise density by a factor of more than 1000 across the entire frequency range. The noise level was reduced from 10–100 pT/√Hz to 0.01–0.1 pT/√Hz (10–100 fT/√Hz) in the frequency range of 0.1–50 Hz. The signal amplitude is up to 120_p-p_ fT for evoked signals from the fetal brain (Eswaran et al., 2005), up to 1200 fT_p-p_ for spontaneous fetal brain activity (Eswaran et al., 2007), >2000 fT_p-p_ for the signals from the fetal heart and the uterus (Wiggins et al., 2013, Eswaran et al., 2002). Thus, the external noise can be reduced to levels sufficiently low to monitor the ongoing activity of the fetal organs in real time.

### 3.2 Rejection of internal magnetic interference

The rejection of internal magnetic field interference depends on the position (x,y,z) and orientation (θ and ϕ) of the noise source relative to the signal dipole and on the coverage angle (Ω) of the sensor array. When the noise dipole is shallow (Fig. 5), the subspace angle (ϖ) between the signal dipole (fetal brain signal source shown by a red sphere) and the noise dipole is independent of sensor coverage angle (Ω) as expected. Fig. 5 is the result for noise dipole 12 located between the brain source and the anterior surface of the abdomen. Generally, the sensor coverage does not affect the subspace angle when the interference is just below the sensor array above or near the brain dipole. The angle depends on θ and ϕ since they affect the orthogonality of the magnetic field produced by each dipole relative to the field produced by the brain source.

When the noise dipole is deeper and/or lateral, ϖ becomes dependent heavily on Ω as well as on θ and ϕ. Fig. 6 shows the results for the interfering dipole #5. This dipole is located to the side (with a large value of ϕ) and relatively deep in the torso (with a large value of θ) to illustrate the usefulness of a full-angle coverage for rejecting interference from internal organs located anywhere in the maternal body. For all values of θ and ϕ, the angular separation becomes larger as the sensor coverage increases from the value of 120°, which is close to the coverage for the SARA system, to the full coverage of 360°, indicating the importance of a full coverage for rejecting the interference from various organs and tissues in all regions of the maternal abdomen.

### 3.3 Imaging of myogenic currents in the entire uterus

Fig. 7 shows the results of the current imaging analysis using the MNE. The active sources (red region) were placed in an anterior and in a posterior region of the uterus closer to the cervix. The current images in the middle row shows the results obtained with the partial coverage array. It can visualize the anterior source, but not the posterior source. The full coverage array, on the other hand, detects both the anterior and posterior activity (bottom). The estimated area of active tissue on the anterior side extends over the actual area. The estimated area is more diffuse on the posterior side. The estimates can be improved by using anatomical constraints as is the case for applications of the MNE for brain sources (Gramfort et al., 2014). For example, the current dipoles could be constrained to be aligned with the direction of the smooth fibers in the myometrium, analogous to the constraint that the dipoles are oriented perpendicular to the cortical surface in the brain.

### 3.4 Detecting ongoing spontaneous activity in fetal heart and brain

**Fig. 8:**
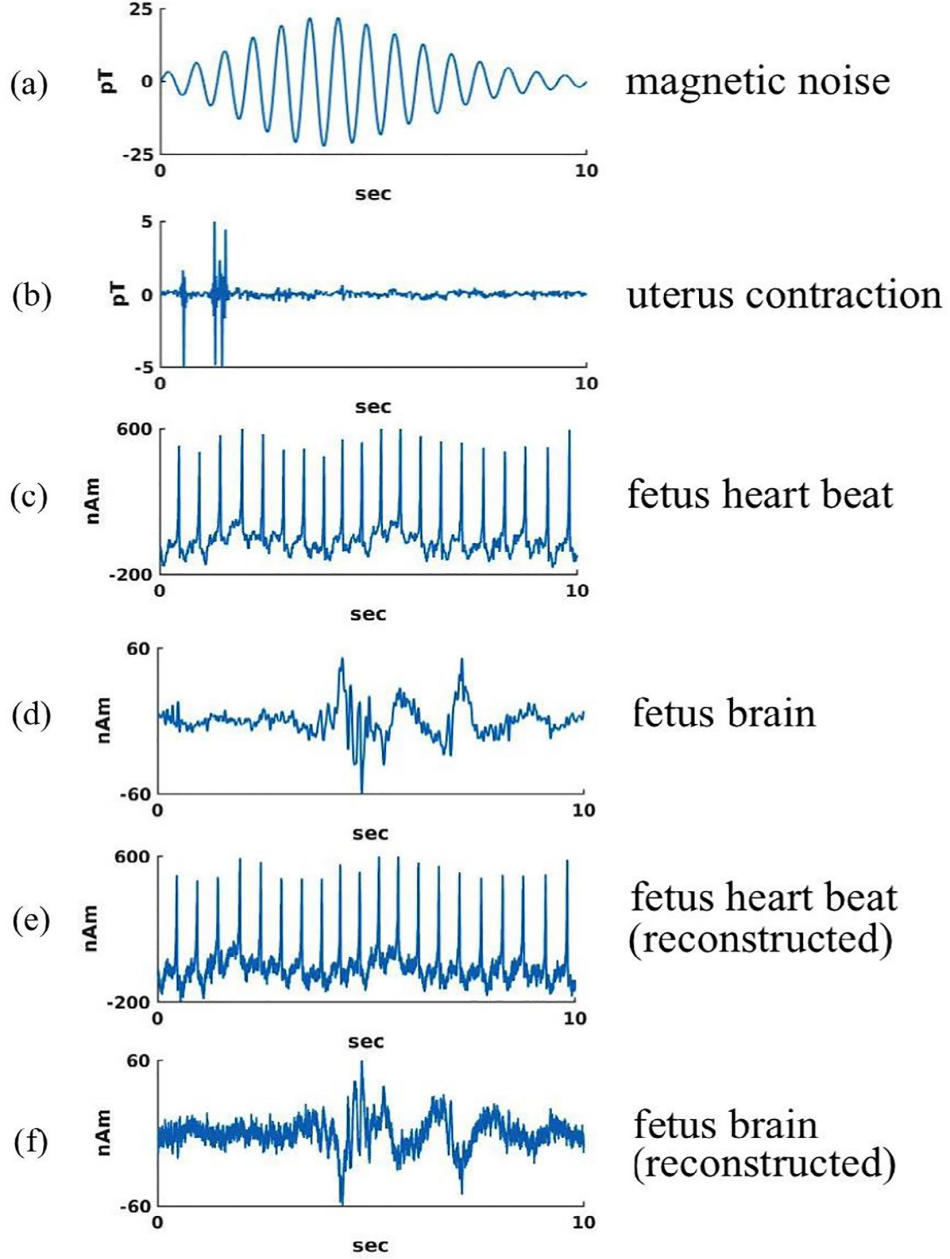
Extraction of fetal heart and brain activity in the presence of an external noise source and internal interference sources. (a) External disturbance. (b) Uterine contraction signal. (c) Fetal heart signal. (d) Fetal brain signal. (e) Extracted fetal heart activity. (f) Extracted fetal brain activity.

Fig. 8 shows the activities of the fetal heart and brain estimated using the beamformer in the presences of various types of magnetic interference. The external noise was a damped sinusoidal waveform due to a moving magnetic dipole 3 m from the model torso (see Method). The uterine contraction is a waveform obtained from the EHG (Alexandersson et al., 2015). The simulated magnetic field was a mixture of the fields from the external noise source, the uterus, and the fetal heart and brain. The bottom two traces (Fig. 8e and f) show the reconstructed source waveforms of the fetal heart and brain using the beamformer. These waveforms have some noise introduced in the reconstruction process, but they are essentially identical to the input waveforms in Fig. 8c and d. The beamformer is thus able to extract the source waveforms of the fetal heart and brain in the presence of the external noise (50 pT peak-to-peak) and the noise from the uterus (10 pT peak-to-peak) that are stronger than the magnetic field from the fetal heart and brain, 5 pT_p-p_ for the QRS complex and ∼1 pT_p-p_ for the brain. Thus, the relatively weak fetal signals are extracted in the presence interference 10–50× stronger.

### 3.5 Detecting cortical activity evoked by external stimulation

**Fig. 9:**
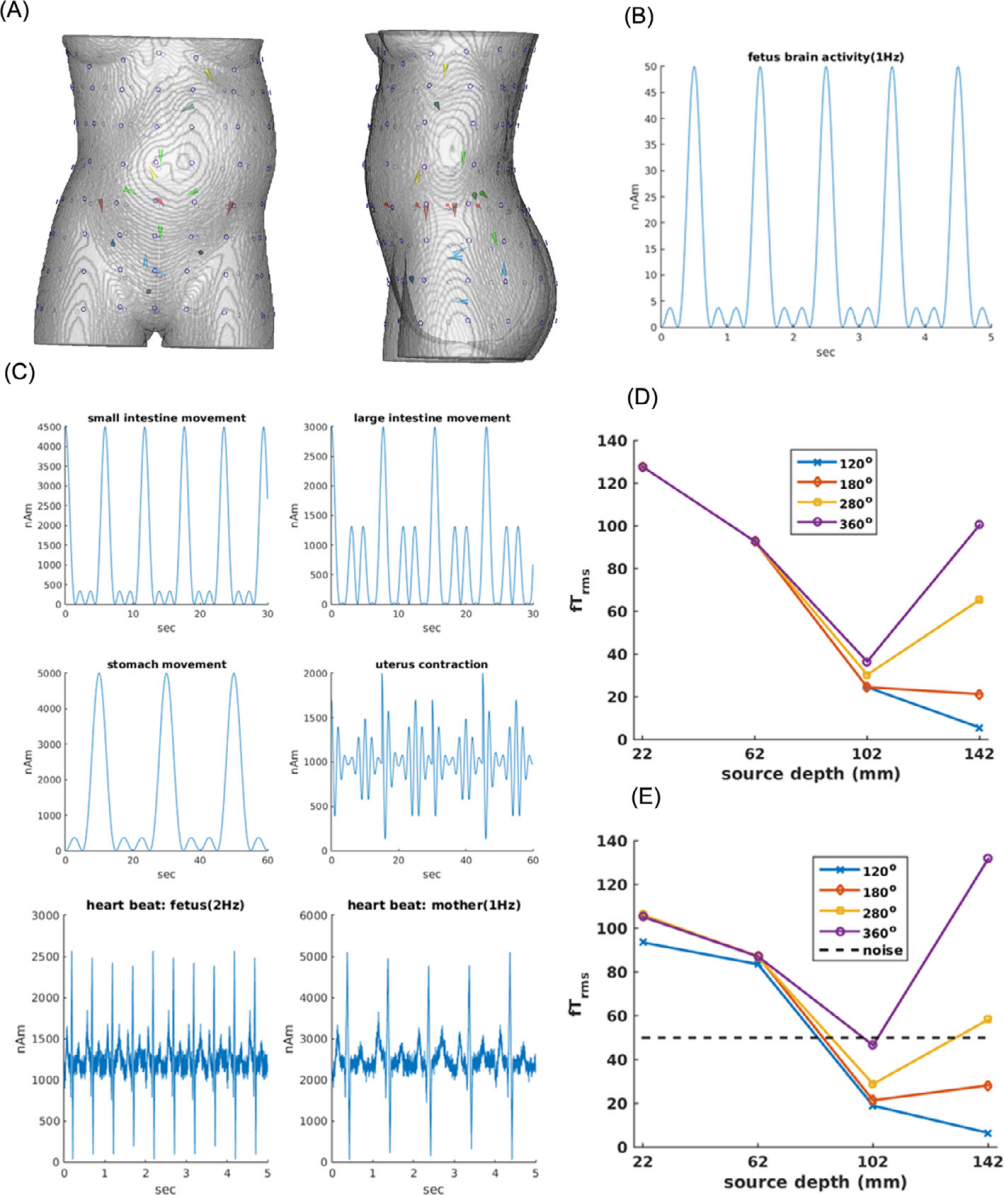
Magnetic field rms strength for a current dipole in the fetal brain as a function of the dipole depth and sensor coverage angle with and without interfering magnetic field noise from internal organs. (A) Signal dipole – a single dipole in the fetal brain (red line with a circle) located at 4 positions along the midline close to the axis passing through the navel. Noise dipoles - a single noise dipole located in the maternal and another in the fetal heart (yellow arrows), a single dipole in the maternal stomach (green, just below the maternal heart source), 5 dipoles in the uterus near the cervix (blue arrows), 2 dipoles in the large intestines (brown arrows on the left and right side on the plane of the fetal brain dipole), and 4 dipoles in the small intestines (light green arrows on a posterior plane centered on the fetal brain dipole). (B) Temporal waveform of the fetal brain dipole (50 nAm maximum). (C) Temporal waveforms of the noise dipoles in the internal organs. Note their moments are 2000–6000 nAm, about 100× stronger than the moment of the fetal brain dipole. (D) Maximum rms field strength at the sensor array for the fetal brain dipole located at 4 depths without any noise field from the internal organs. (E) Same with the interfering magnetic field from the internal organs. Dotted line indicates the noise level of the simulated OPM sensors in fT_rm_. (For interpretation of the references to colour in this figure legend, the reader is referred to the web version of this article.)

The capability for detecting cortical evoked activity was evaluated for sensor arrays varying in angular coverage as a function of depth of the fetal brain source. The rms strength of the signal from the dipolar fetal brain source was calculated as a function of the dipole depth without any internal noise source and in the presence of these noise sources. Fig. 9A shows the four dipoles (red) on the anterior-posterior axis passing through the navel at depths of 22, 62, 102 and 142 mm below the abdominal surface. The thickness of the torso was 195 mm at this level. Fig. 9B shows the temporal waveform of this dipole in the brain. Its dipole moment (Q) was 50 nAm consistent with the literature. Fig. 9D shows the rms field strength at the sensor with maximum signal without the internal noise sources. There is no effect of the coverage angle when the dipole is close to the abdominal surface, but the coverage has greater effects for deeper dipoles. The signal is minimum close to the center of the abdomen (98 mm). The signal continues to decrease as the signal dipole becomes deeper for the sensor arrays with small coverage angles (120° and 180°). It increases for the sensors with wider coverage as the dipole approaches the posterior abdominal surface because the signal dipole is closer to the posterior abdominal surface (depth of 142 mm from the anterior surface = 53 mm from the posterior surface). Fig. 9E shows the results in the presence of noise sources in the key internal organs (maternal heart, uterus, stomach, small and large intestines and fetal heart). The temporal waveforms of the interfering noise sources are shown in Fig. 9C. Their dipole moments are about 100 times larger than the moment of the fetal brain source. Nevertheless, the SSP is able to suppress most of these strong noise sources and extract the signal from the brain. There are some notable effects of the noise (i.e. for the shallow source at depth of 22 mm and the deepest source at depth of 142 mm). Overall, however, the extracted amplitude behaves similarly to the case without the noise sources (Fig. 9D) as a function of source depth and sensor coverage.

These results demonstrate that the full-coverage sensor array is superior to the partial-coverage sensor arrays. The signal level for the full-coverage array is 1–2× the noise level expected for the OPM instrument (10 fT/√Hz, 25 Hz bandwidth = 50 fT) even when the brain is deep (∼5–10 cm from the anterior or posterior side). The SNR can be, therefore, >10:1 if 100 evoked responses are averaged. Evoked cortical responses can be thus measured with a SNR of close to 10:1 in ∼3 min with a presentation rate of one stimulus every 2 s. This indicates that it should be possible to study cortical activity elicited by external stimulations within a reasonable period of time using a full-coverage FM scanner.

## 4 Discussion

This study evaluated the possibility of noninvasively measuring ongoing electrophysiological activity of the uterus and fetal organs with a biomagnetic technique. Overall, we showed that it should be possible to monitor the activity online in real time using a novel type of biomagnetic instrument that provides a full coverage for measuring the magnetic field from the chest to the hip area of a pregnant mother. The online monitoring capability would provide an important new power in analysis and diagnosis important for maternal and fetal health care since the results could be used for immediate feedback in optimizing the monitoring procedure or interventions. The intervals between the QRS’s in fetal ECG can be used to estimate the instantaneous heart rate, which is useful for monitoring cardiac arrhythmia (Taylor et al., 2003). It is possible that the improved rejection of the interference from the external and internal noise sources improves the SNR sufficiently to monitor the P-R and S-T intervals reliably in real time from the fMCG. These intervals are essential for predicting potential life threatening cardiac events in the fetus. Similarly, real-time monitoring of the uterine activity during the quiescent period before the labor starts should be valuable in predicting the onset of preterm and term labor. Real-time monitoring of fetal brain activity is valuable for detecting the presence of pathophysiological spontaneous activity that, for example, can reveal the presence of epileptiform activity due to cortical dysplasia or genetic or metabolic abnormality. Below we discuss some of the key issues in developing this type of technology.

### 4.1 Rejection of external magnetic disturbance

One of the major problems in detecting the magnetic fields from internal organs of a pregnant woman is rejection of external magnetic interferences. Conventionally, this is accomplished by measuring the magnetic field inside a magnetically shielded room (MSR) (Vrba et al., 2004, Sheridan et al., 2010). An MSR reduces the magnetic field inside using layers consisting of a hypermalloy (alloy with a very high magnetic permeability with a relative permeability μ of >50,000–100,000) and aluminum. To increase the shielding factor, the magnetic field inside can be reduced further by an active shielding method. We have implemented such a method for our MEG facility at Boston Children’s Hospital (Okada et al., 2016). The active shielding reduces the field inside by monitoring the magnetic field just outside or inside the MSR along the three orthogonal axes and using this information in a feedback circuit. Together, the disturbance can be reduced to approximately 10 nT at DC and <0.1 nT in the low frequency range below 1 Hz. The magnetic noise must be reduced further in order to measure the signals from the fetal heart and brain reliably and clearly. Thus, additional noise rejection techniques need to be used. In the present case, we have shown that the technique called SSP can reduce the noise by a factor of ∼1000 when it is used with the full-coverage array. If the passive and active shielding methods can reduce the AC noise to about 100 pT, this SSP can reduce the noise level further to 100 fT, which is smaller than fMCG signals and comparable or lower than spontaneous fMEG signals. Then, fMEG as well as fMCG can be measured clearly with the FM scanner.

The use of a large MSR is costly and has inhibited the popular use of biomagnetic methods for clinical applications. In the present study, we considered an alternative, much more economical method of magnetic shielding, using a cylindrical shield that can fit into an enclosure that looks like a small MRI scanner. A 3-layer mumetal cylindrical shield has been used for an atomic magnetometer system (Xia et al., 2006). The FM scanner based on the OPMs is not interfered by radio-frequency signals unlike the conventional biomagnetic detectors based on superconducting quantum interference devices (SQUIDs). Thus, an FM scanner can be placed in any ordinary room in a clinic without the expensive electromagnetic shielding.

### 4.2 Rejection of internal biological sources of magnetic interference

In addition to rejecting the external interference, the magnetic field from various internal organs must be separated in order to detect the signal from an organ of interest. We have shown that our full-coverage sensor array design is superior to partial-coverage sensor arrays with a varying degree of coverage around the torso. The existing FM scanners such as SARA provide a coverage of about 110° or less around the torso (Vrba et al., 2004). As was shown in Fig. 6, the subspace angle between the field of a brain dipole and the field of an internal noise dipole is wider when the sensor coverage is wider, especially for deeper sources, with a maximum subspace angle for the full-coverage sensor array. The full-coverage sensor array thus provides superior rejection of the interference from internal organs as well as the external noise sources for clearly measuring the activity of the fetal heart and brain.

### 4.3 Detection of the activity of the uterus

The activity of the uterus is important for understanding the preterm and term labor. The physiology of uterine contraction has been studied in animal models (Maul et al., 2003). The mechanical contraction of the uterus is commonly measured with tocodynamometry (Bakker et al., 2010). The electrophysiological activity of the uterus, including increase in synchronization of the myometrial activity and propagation of the contractile activity, can be measured either electrically (Buhimschi et al., 1997, Terrien et al., 2010, De Lau et al., 2013) or magnetically (Eswaran et al., 2002, Ramon et al., 2005, Escalona-Vargas et al., 2015, Govindan et al., 2015). Although they are quite useful, these analyses have been limited to the sensor level.

We have shown that currents in the uterine wall at the source level can be visualized. The full coverage array could visualize the activity on the anterior and posterior sides of the uterus, whereas a partial coverage array could visualize the activity only on the anterior surface, but not on the posterior surface. This result suggests that the full-coverage FM scanner may be used to visualize the activity in the entire uterus. We still do not know the sensitivity of this technique. However, it is worthwhile exploring the possibility of detecting the myogenic activity during the relative quiescent period between contractions for understanding the development of synchronized activity preceding the onset of contractions and labor or even during the menstruation cycles. If the activity can be detected clearly during the quiescent period, one could try to visualize the initiation site and the propagation pattern.

The measurements of the uterine activity can be combined with mathematical models of the electrophysiology of the uterus being developed by several groups (LaRosa et al., 2012, Yochum et al., 2016) for increasing our understanding of the physiological bases of the signals.

### 4.4 Detection of activity of fetal heart

Our simulation study indicates that it might become possible to detect the fetal MCG online. The fetal MCG signals can be detected with the existing FM scanners (Cuneo et al., 2009, Strasburger and Wakai, 2010). However, the detection of the fetal MCG is still very difficult and the analysis is offline since the interference from the maternal heart and other organs must be removed in addition to the external noise. We do not know of any biomagnetometers that can detect the fetal MCG online. It would be important to validate our prediction of the new online capability with a full-coverage array.

Echocardiography cannot detect the electrical activity during the mechanically silent period such as the ventricular repolarization phase (Wiggins et al., 2013). Neither ultrasound nor ECG can detect the details of the electrical events during each cardiac cycle such as the P-R duration, the shape of the onset of the QRS (important for diagnosing the Wolff-Parkinson-White (WPW) syndrome), and the S-T duration (important for long QT syndrome) and T amplitude (important for detecting the T alternans). These parameters are critical for detecting and diagnosing cardiac arrhythmias in the fetus, which are essential for predicting and preventing premature delivery, still death and postnatal heart problems. It would be important to test whether the full-coverage would enable online detection of the P-R and S-T intervals as well as the R-R interval, since this capability, if reliable, could enable the detection of cardiac arrhythmia in the fetuses in utero.

### 4.5 Detection of activity of fetal brain

In addition to detection of fetal heart activity, our simulation study indicates that it might become practical to detect the ongoing spontaneous activity of the fetal brain in utero. This capability, if validated, would represent a major advance in obstetrics and fetal medicine (Sheridan et al., 2010). Our results indicate that the spontaneous activity could be isolated from the magnetic fields produced by a strong external disturbance and the uterus. Amplitudes of spontaneous brain rhythms are close to 500 fT, which gives an SNR of 10:1 for the noise level of 50 fT expected for measurements with a bandwidth of 25 Hz for a 10 fT/√Hz OPM system.

It might also become possible to detect evoked cortical activity from the fetal brain with a relatively small number of averages over a wide range of head positions. The detection of evoked brain activity in the fetus is a big challenge today (Sheridan et al., 2010). It may become much easier to measure evoked brain activity with our approach. The signals for a current dipole with a dipole moment Q of 50 nAm are about 100 fT, which gives an SNR of 2:1 for an FM scanner with OPMs with a noise level of 50 fT (field sensitivity of 10 fT/√Hz, recording bandwidth of 25 Hz). Thus the SNR can be ∼ 10:1 after averaging as few as 25 responses. The signal was above 50 fT over a wide range of head positions for the full coverage sensor array. This capability, if validated with an actual FM scanner, could be useful in basic developmental neuroscience.

## 5 Conclusions

Based on the performance seen in this simulation study, we predict that it will become possible to monitor the ongoing electrical activity of the uterus and fetal brain and heart in real time using a full-coverage FM scanner. The full-coverage array is useful for reducing the magnetic interference from external noise sources to a level close to the instrumentation noise level. It is also useful for rejecting the magnetic interference from internal organs throughout the torso of the mother to clearly detect the electrophysiological activity of the internal organs of interest. We anticipate this approach could become very useful as a new clinical modality in clinical neurophysiology and fetal and maternal medicine.
